# Diagnostic Tests to Support Late-Stage Control Programs for Schistosomiasis and Soil-Transmitted Helminthiases

**DOI:** 10.1371/journal.pntd.0004985

**Published:** 2016-12-22

**Authors:** Kenneth R. Hawkins, Jason L. Cantera, Helen L. Storey, Brandon T. Leader, Tala de los Santos

**Affiliations:** Diagnostics Program, PATH, Seattle, Washington, United States of America; University of California Berkeley, UNITED STATES

## Abstract

Global efforts to address schistosomiasis and soil-transmitted helminthiases (STH) include deworming programs for school-aged children that are made possible by large-scale drug donations. Decisions on these mass drug administration (MDA) programs currently rely on microscopic examination of clinical specimens to determine the presence of parasite eggs. However, microscopy-based methods are not sensitive to the low-intensity infections that characterize populations that have undergone MDA. Thus, there has been increasing recognition within the schistosomiasis and STH communities of the need for improved diagnostic tools to support late-stage control program decisions, such as when to stop or reduce MDA. Failure to adequately address the need for new diagnostics could jeopardize achievement of the 2020 London Declaration goals. In this report, we assess diagnostic needs and landscape potential solutions and determine appropriate strategies to improve diagnostic testing to support control and elimination programs. Based upon literature reviews and previous input from experts in the schistosomiasis and STH communities, we prioritized two diagnostic use cases for further exploration: to inform MDA-stopping decisions and post-MDA surveillance. To this end, PATH has refined target product profiles (TPPs) for schistosomiasis and STH diagnostics that are applicable to these use cases. We evaluated the limitations of current diagnostic methods with regards to these use cases and identified candidate biomarkers and diagnostics with potential application as new tools. Based on this analysis, there is a need to develop antigen-detecting rapid diagnostic tests (RDTs) with simplified, field-deployable sample preparation for schistosomiasis. Additionally, there is a need for diagnostic tests that are more sensitive than the current methods for STH, which may include either a field-deployable molecular test or a simple, low-cost, rapid antigen-detecting test.

## Introduction

The World Health Organization (WHO) has issued a roadmap for control, elimination, or eradication of 17 neglected tropical diseases (NTDs) by 2020 [1]. An international consortium of public and private sector partners have signed the London Declaration on NTDs, pledging specific efforts against ten NTDs [2] including schistosomiasis and soil-transmitted helminthiases (STH), which are both caused by parasitic worms.

Schistosomiasis is a debilitating disease resulting from infection by trematode worms of the genus *Schistosoma* [3,4]. The species that cause most of the morbidity are *Schistosoma haematobium* (Africa), *S*. *mansoni* (Africa and South America), and *S*. *japonicum* (Asia).

STH diseases result from intestinal infections of nematode worms, including the large roundworm *Ascaris lumbricoides*, the whipworm *Trichuris trichiura*, and two hookworm species, *Ancylostoma duodenale* and *Necator americanus*.

Details of the natural history and life cycles of schistosomes and STH have been described in numerous publications.[5–7] *Schistosoma* spp. spend part of their life cycle in cognate planorbid snail intermediate hosts. Tools to monitor the prevalence of infection in snails are therefore important; however, we have confined our scope to human diagnostics.

Estimates of morbidity and mortality and guidelines for control programs on determining regional prevalence and determining treatment are comprehensively described in WHO publications for both diseases [8]. The WHO goal for both schistosomiasis and STH is disease control and reduction of morbidity, to be achieved by a national coverage rate of 75% of the targeted communities with praziquantel (PZQ) [3] and mebendazole and albendazole for STH. For schistosomiasis, regional elimination is now also a goal [9].

The public sector and private pharmaceutical companies have contributed significantly to schistosomiasis and STH control programs through large-scale donations of drugs used for treatment. Monitoring the effectiveness of these efforts relies on the accuracy of WHO-approved diagnostic methods, which currently consist of microscopic examinations of stool or urine samples for parasite eggs. However, better diagnostic tools will be needed to inform decisions to reduce or stop mass drug administration (MDA) as treatments are scaled up and prevalence begins to decline. In the absence of more sensitive and robust diagnostic tools, disease prevalence estimates which are used to inform decisions regarding use of MDA will remain limited in their accuracy. Incorrect decisions that result in the over- or underuse of MDA ultimately comes with costs to donors, control programs, and affected populations.

In this paper, we review diagnostic needs for schistosomiasis and STH control programs, assess the potential of new tests, and determine appropriate strategies for improving diagnostic testing to support the goals for these two diseases laid out in the WHO 2020 roadmap [1].

## Methods

Between August and December 2014, we gathered input from representative international stakeholders, including disease experts, laboratory and field researchers, test developers, program implementers, mathematical modelers, policymakers, and donors. In parallel with stakeholder analysis, we conducted a review of publicly available literature and manufacturer websites to assess diagnostic needs, identify potential solutions, and determine an appropriate strategy for diagnostic testing to support disease elimination efforts. We used an exploratory search strategy for both websites and public databases including PUBMED and MEDLINE, starting with known entry points (reviews, key stakeholders, etc.) and allowing those interactions to inform the next cycle of search terms and contacts in a cascade search. We judged the search to be complete when saturation was reached. Key publications are noted in the Top Five Papers box.

## Findings

### Current diagnostic tests

The current gold standard method for determining the presence of *Schistosoma* parasite eggs is microscopic examination of stool and urine samples [9]. The Kato-Katz method [10] is the most common preparation for copromicroscopy (stool microscopy), and uroscopy (urine microscopy) is also used, after filtration of urine. However, neither method is sensitive to the low-intensity infections that characterize populations treated with PZQ. A rapid diagnostic test (RDT) for the schistosome excretory/secretory (ES) circulating cathodic antigen (CCA) has received considerable attention recently and is now recommended as an alternative to Kato-Katz for *S*. *mansoni*, but it is insensitive to *S*. *haematobium*, and validation has not been demonstrated for other species [11–20].

For STH, the gold standard diagnostic test is also Kato-Katz copromicroscopy, which is also used to distinguish helminth eggs for each of the four STH species. Helminth eggs are not excreted in urine, so uroscopy is not used for STH. Another copromicroscopy method, the mini-FLOTAC, has been recommended recently for use in STH surveillance by WHO and may improve specimen preservation and the ease of slide reading, although it requires specialized test materials for sample collection and analysis, adding to cost and complexity [21]. A recent study [22] compared these copromicroscopy tests for detecting STH using Bayesian latent class analysis in the absence of perfect reference standard. Their data suggested that the most commonly used double slide Kato-Katz method had a sensitivity of 74%–97% at high STH infection intensity, with a reduction in its sensitivity to 53%–80% in low intensity settings. This is similar to the sensitivity of the mini-FLOTAC method. The FLOTAC has the highest sensitivity in both infection intensities (69%–86% and 97%–99% for low and high intensities, respectively). Although the sensitivity of copromicroscopy methods is sufficient for informing early stages of control, it is inadequate when infections are reduced to low levels by MDA.

### Use cases for improved tests: reducing or stopping MDA and post-elimination surveillance

**Fig 1:**
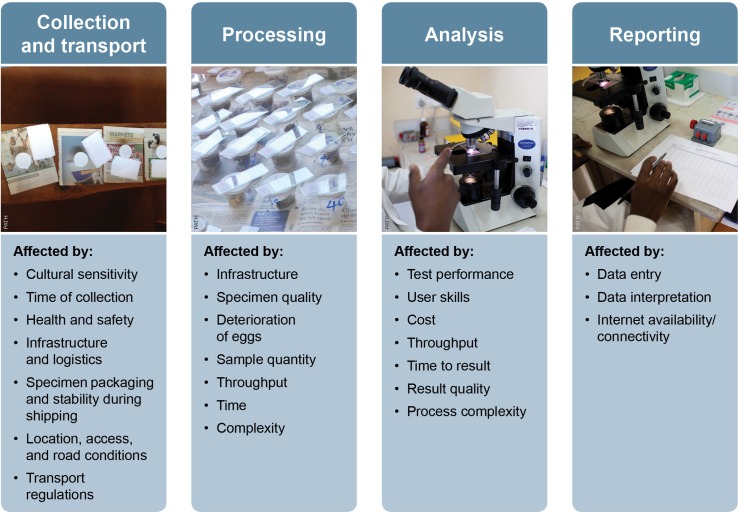
The steps required for gold standard microscopy in deworming programs. In the typical surveillance testing performed to assess the prevalence of helminth infection and the impact of deworming programs, stool samples (or sometimes urine for schistosomiasis) are collected and transported to a nearby laboratory space for microscopic analysis and follow-on reporting. There are numerous factors affecting each step of the process that contribute to making this analysis less than optimal.

Control programs based on MDA have four designated stages: mapping disease prevalence, monitoring the impact of MDA interventions, making decisions to reduce or stop MDA, and performing surveillance after elimination has been certified [23]. The current microscopy-based diagnostic tools for schistosomiasis and STH are suitable for addressing testing needs for the first and second stages by using methods that count the number of parasite eggs excreted in urine or stool. The main strengths of this type of tool are extensive validation and familiarity worldwide. For schistosomiasis, the egg counts are also a better proxy for disease (as opposed to infection), since the primary cause of morbidity is the damaging effects of the eggs migrating through tissue—further bolstering the use of microscopy (and other morbidity markers) in the first and second stages of control programs [24]. Because the operational requirements of microscopy-based testing are relatively modest, the technique can be used at lower levels of the health system (Fig 1). A major limitation is insufficient sensitivity for detecting infections that have a low burden of parasites, which diminishes usefulness in later disease control stages. While the presence of eggs in excreta is a good proxy for morbidity at high worm burdens, it is not the best proxy for future transmission risk with lower worm burdens [25]. For example, recent work has shown that reliance on egg counting to infer efficacy of PZQ dosing in intestinal schistosomiasis has resulted in systematic under-dosing and concomitant failure to clear all worms from a pediatric Ugandan population [26]. In addition, although the Kato-Katz technique is generally considered a low-cost method, a “true cost” analysis (including all personnel required and the time it takes to administer the program) suggests the overall cost is much higher than previously thought—about US$2.00 per child for a single Kato-Katz per child, the least sensitive embodiment. In comparison, the current CCA test costs about US$2.20 (assuming US$1.75 for the test and US$0.50 program costs) [13].

Control programs for schistosomiasis and STH need accurate surveillance for the third and fourth control program stages to inform decisions about reducing and stopping MDA and to conduct postelimination surveillance once elimination is certified. In both of these use cases, the diagnostic tools must be more sensitive than the current microscopic methods, which can miss eggs because of low parasite burden after MDA and because of naturally occurring variability in samples, even from the same individual. As more countries move toward reducing prevalence and reaching elimination goals, the ability to identify and target reservoirs of infection that persist or reemerge could expedite global elimination goals and facilitate the subsequent winding down and conclusion of massive, costly drug donation and disease control programs. The emphasis in the late stages should be on tools that detect infection, even in the absence of disease.

### Developing new diagnostic tools

#### Basic considerations

In developing new diagnostic tools, researchers must make decisions about biomarkers and platforms—the type of test that will support detection of the biomarker while meeting operational requirements of the control program. Biomarker properties that target specificity, biological uniqueness, and abundance and persistence in accessible (and acceptable) sample types need to be considered (Table 1). Many biomarkers may be applied to multiple platforms (e.g., antigens can be applied to both enzyme-linked immunosorbent assays [ELISA] and immunochromatographic RDTs), but if a biomarker is not unique to the parasite under investigation, or if people are unwilling to collect the types of specimen required, no platform will make it useful. In regard to biomarkers, tests can be direct or indirect. In general, direct tests (interrogating the presence of the parasite) are more appropriate for informing MDA as prevalence and infection intensity fall, because they detect actual infection. Indirect tests (interrogating the host response) are more appropriate in postelimination use cases because they detect exposure, which should be absent postelimination [27].

**Table 1:** Overview of STH and Schistosomaiasis (SCH) diagnostic landscapes.

Biomarker	Surveillance Measure	Description of Putative Biomarker Candidates	Sample Type	Format	Examples in STH or SCH Diagnostics	Stages in Product Development	Identified Use Cases
Parasite	Infection	• Eggs	• STH, SCH: Stool • SCH: Urine	Microscopic exam	• Kato-Katz • McMaster • Mini-FLOTAC • Urine filtration	• Developed, commercial products available and in common use	• Mapping • Impact monitoring
Parasite proteins	Infection	• STH: ES; somatic proteins • SCH: circulating anodic antigen (CAA), CCA; soluble egg antigens (SEAs); ploycomb group protein (PcG), RAD23	• STH: Stool • SCH: Blood, urine, stool, saliva	Antigen detection immunoassay	• RDT • ELISA	• STH: Research and development; no commercial diagnostic product currently available • SCH: Commercial product either available (CCA-RDT) or in development (CAA-RDT, CAA-UCP, RDT)	• Mapping • Impact monitoring • MDA-reduction decision • Post-MDA surveillance
Parasite nucleic acid	Infection	• STH, SCH: rRNA genes, internal transcribed spacer (ITS); mDNA (*cox1*); high copy number, noncoding repeat DNA sequence • SCH: Dra1 tandem repeat; miRNA223	• STH: Stool • SCH: Blood, urine, stool	Lab- or field-based molecular tests	• Conventional polymerase chain reaction (PCR) • Real-time PCR (multiplex of multiparallel) • Isothermal amplification assay	• “Homebrew” PCR assays available for research use only • Limited field demonstrations	• Mapping • Impact monitoring • MDA-reduction decision • Post-MDA surveillance
Antibodies against parasite antigens	Exposure	• STH: α-ES, α-L3 • SCH: α-SmCTF, α-AWA, α-SEA, α-CEF6, α-SBgA	• STH, SCH: Blood	Antibody detection immunoassay	• RDT • ELISA	• STH: Research and development; No commercial product available • SCH: New products in development (α-SmCTF RDT)	• Post-MDA surveillance

Table 1 provides an overview of STH and *Schistosoma* diagnostic landscapes.

New target product profiles (TPPs) for tests to detect various biomarkers in specific use cases for both schistosomiasis and STH have recently been developed and are publicly available at http://sites.path.org/dx/ntd. TPPs have been constructed for lateral flow tests for antigen biomarkers and for nucleic acid amplification tests (NAATs)—both intended to inform decisions to adjust or stop MDA. A TPP was also developed for lateral flow tests for antibody biomarkers, for the use case of postelimination surveillance. The TPPs define acceptable and ideal requirements for test characteristics such as intended use, stability, specimen type, throughput, and performance, including clinical sensitivity and specificity. The **ASSURED** (**A**ffordable, **S**ensitive, **S**pecific, **U**ser-friendly, **R**apid and **R**obust, **E**quipment-free, **D**elivered to those who need it) criteria [28] were considered at all times during TPP development to align test characteristics with their intended use within low resource settings.

Control programs for both schistosomiasis and STH have historically focused monitoring efforts on school age children. Decisions are made about regional MDA based on prevalence and treatment efficacy surveys on sentinel schools. As prevalence drops and elimination is approached, the population sample should be expanded to include younger children and adults to ensure accurate tracking of infection and morbidity and precision in the administration of treatment [26]. The expansion of the sample frame and declining prevalence may also change the mode of testing from the current paradigm of community diagnosis though the sentinel schools, toward individual diagnosis. All of these factors influence the TPPs of new diagnostic products for attributes like robustness, throughput, training of the user, and ancillary equipment. The applicability of the ASSURED criteria will also change with the changing requirements. An excellent discussion of the topics in this paragraph on schistosomiasis in Africa and Arabia has been published previously [29].

Ultimately, the optimal design of a diagnostic tool will be decided by field user feedback and operations research with actual products or prototypes. Thus, TPPs are living documents that require updates as new data is gathered and user needs change.

#### Schistosomiasis

Ideally, a new diagnostic test for schistosomiasis will offer improved accuracy and operational characteristics sufficient to justify the transition costs to control programs from current microscopic methods [7]. Low limit of detection (LOD) requirements for the new test can be addressed by improving the signal-to-noise (S/N) ratio in several ways, including preanalytical concentration of analyte from a large sample volume, preanalytical enzymatic amplification of analyte or use of high S/N labels (fluorophores or up-converting phosphors), and the use of instruments to reduce noise and eliminate bias [30–44].

Key opinion leaders from the Schistosomiasis Consortium for Operational Research and Evaluation (www.score.uga.edu) have concluded that a sensitive RDT for a circulating antigen constitutively produced by a fecund adult worm pair is the most promising tool for development. Researchers at Leiden University Medical Center (LUMC) have already identified suitable antigens [32, 43, 45–48]; and an RDT for one of them, CCA, was successfully commercialized and is becoming widely adopted. However, CCA has only been shown to be useful for *S*. *mansoni* [11, 14–20] and also has a potential for cross-reaction with other parasite antigens and some human cancers [49,50].

Diagnostics using CAA have also been developed and evaluated extensively, including field demonstrations [32–33, 40–41]. CAA is a polysaccharide waste product of adult worm pairs that is present in several accessible sample types (blood and urine), very stable, and genus specific (one product can guide MDA in all endemic countries, regardless of the geographic distribution of *Schistosoma* species). This work has established CAA as the preferred analytical target. The current, most sensitive embodiment of the LUMC CAA RDT uses a preanalytical concentration step, up-converting phosphor particles, and a reader instrument in a lateral flow strip format. While this combination has demonstrated excellent performance with a LOD for the most sensitive version corresponding to a single worm pair [33], it does not yet meet TPP optimal operational requirements in terms of ease of use and throughput, primarily due to the format of the preconcentration step. Translation of the preconcentration step to a method that is more field-deployable and is better matched to the workflow of the RDT will be required to address field surveillance use cases.

Another advantage of the antigens identified by LUMC is that the antigens have been characterized with an experimental primate model that allows correlation to actual adult worm burden and, therefore, good estimation of how serum or urine concentration of the antigen relates to the parameter of most interest to elimination programs (low number of fecund worm pairs) [51]. Correlation of the antigen concentration to egg counts when the fecund worm burden is low is difficult, because the LOD of Kato-Katz is too high to serve as a good reference in this worm burden regime. This is especially true after MDA when some of the worms may not be producing eggs, but are still viable, and may recover to lay eggs again. The ability of these excretory antigens to signal the presence of worms even when the primary source of morbidity (eggs) has been abolished makes them uniquely suited to elimination use cases; although, they can also be used to stratify morbidity risks at higher worm burdens [26].

Other diagnostic tests that may eventually prove valuable adjuncts to CAA testing in the postelimination use cases include antibody tests and new molecular diagnostic tests targeting *Schistosoma* DNA. Several antibody tests have been described and with further development or and/or validation could have postelimination application [52–54]. Molecular tests, which use a preanalytical enzymatic amplification to achieve sensitivity, appear to have high potential but have not as yet been developed into commercial human diagnostic products or adapted for point-of-care (POC) use. PCR for a variety of targets has been investigated the most [35, 55–69], but several isothermal methods have also been described [44, 70–71]. Advantages and limitations of molecular methods for schistosomiasis are similar to those for STH, discussed below, with the exception that stool may not be the only suitable sample. The ongoing emergence of new POC molecular platforms and the potential for multiplexing with other NTDs suggests that molecular tests will become increasingly important. They are also the most likely candidates to adequately address detection of infection in the snail intermediate hosts, as adult worms (and their antigens) are not found in the snails.

#### STH

The situation for STH is very different from that for schistosomiasis, because there is no validated STH antigen that acts as a biomarker for developing a new diagnostic test. Based on their current stage of development, including the availability of well-characterized genomic biomarkers and assay protocols that have undergone initial verification, NAATs for STH are promising [72–82]. PCR-based approaches have demonstrated improved performance over microscopy for detection of STH species and allowed simultaneous analysis of multiple STH targets in parallel or through multiplexing [83–85]. However, as with molecular methods for other infectious diseases, the use of real-time PCR assays for STH are limited to research or use by a limited number of country programs with the necessary infrastructure and adequate resources such as expensive and specialized equipment, reagents, and trained personnel. Nonetheless, the potential sensitivity improvements over microscopy, the availability of characterized genomic targets, and the potential to add additional targets such as those for other important enteric pathogens make real-time PCR an attractive option for STH detection. Currently, there is no molecular assay for STH diagnosis that has been adapted into a commercialized product, and the STH research community continues to advance numerous lab-developed assays toward a single-reference standard protocol for STH detection.

For new tests to be viable alternatives for current methods, they must have performance characteristics that make them suitable replacements. This includes the ability to detect and distinguish each of the four high-priority STH species—which is important for selection of drugs to use in MDA programs—and to quantitate the intensity of infection. Further research may be required to understand the correlation of DNA levels with numbers of live worms or eggs and the impact that DNA from nonviable worms may have on assay interpretation, including prevalence and intensity estimates. From an assay design standpoint, further research is needed to ensure assays can provide robust, reliable quantification of STH genomic targets and to determine how quantitative NAAT results will be developed into guidelines as an alternative indicator to counts of helminth eggs for assessing infection intensity. Once STH molecular assays have been validated, protocols will likely require further refinement to adapt lab-developed methods for use in commercial tests, including those intended for use with field-deployable molecular platforms that may be needed to ensure access for all STH programs.

Stakeholders have indicated a preference for a low-cost and sensitive RDT for assessing parasite antigens, as it could potentially be performed immediately onsite during sample collection and reduces requirements for specialized training associated with current microscopy-based methods. To date, however, there are limited data validating STH antigens for use in such tests, particularly for a complete set of biomarkers that would allow detection of all four major STH species either alone or in concert. Results of a limited number of early research studies have identified candidate antigen biomarkers such as the ES proteins that may be of use in detection of different STH species. Research [86–89] must ensure that new antigen markers offer adequate specificity and do not cross-react with analogous proteins in other related helminth species found in the same environment. Although lab-based platforms such as ELISAs would likely accommodate multiplex immunoassays if suitable biomarkers are identified, stakeholders expressed a preference for a multiplex test that is field-deployable. New POC immunoassay platforms are emerging that may accommodate the multiplexing needed for pan-STH detection, including some newer RDT formats that may not require the use of a separate reader. Further validation of emerging biomarkers is needed.

Development of improved specimen collection tools is also an important pathway to new diagnostic tests. Stool is currently the only validated sample due to the biology of the STH infection. Control programs would prefer blood or urine samples for diagnostic testing, but that will require further research to identify and assess the feasibility of alternative STH biomarkers in other matrices such as blood or urine. If stool remains the only feasible sample for STH diagnosis, additional research is needed to improve specimen collection methods, handling, and processing techniques. These include evaluation of alternative stool collection methods, such as Bio-wipes [90] or rectal swabs [91], that may be more acceptable to users.

Tables that detail the comprehensive landscape of biomarkers and technologies for both schistosomiasis and STH are attached as electronic supplementary information (S1–S4). Key learnings are summarized in the Key Learning Points box.

## Conclusions and recommendations

The lack of appropriate diagnostic tools to support late-stage decisions for MDA of schistosomiasis and STH remains a serious gap in disease control and elimination programs. Microscopic methods to count parasite eggs will remain the WHO-recommended method for schistosomiasis and STH surveillance until evidence is generated that clearly demonstrates how diagnostic tools measuring alternative indicators—infection and exposure—will significantly improve decision-making by control programs. Currently, key scientific advisors to WHO continue to prefer microscopy due, in part, to its perceived low cost; demonstrated efficacy in high-prevalence, high-worm burden settings; and longstanding use by control programs.

If performance requirements can be met, our review suggests that field-deployable, highly sensitive, and specific RDTs for unique parasite antigens are the preferred solution for supporting decisions to adjust community MDA programs based on cost and familiarity with such a format. Postelimination surveillance may be supported by similar POC tests for antibody biomarkers. Preferably, these new diagnostics should cost programs no more that approximately US$2/child to purchase and use—more expensive tests may not be adopted due to concerns about extra costs relative to microscopic techniques.

### Schistosomiasis

Our findings lead to several conclusions and recommendations specific to the development of new diagnostic tests for schistosomiasis. First, the analyte should be CAA. The CAA test is preferred because of the advanced state of CAA characterization, current availability of commercialized platforms, and strong advocacy among key stakeholders. If other tests are developed, prioritized biomarkers would include an antibody. Molecular markers may become more important as POC platforms become more practical and available. Second, RDTs based on lateral flow immunochromatographic strips using highly sensitive labels and a cognate reader to increase S/N ratios will provide adequate sensitivity for low-intensity infections while retaining desired ease of use. Finally, field-deployable POC CAA preconcentration methods are needed for the lowest worm burden use cases (elimination certification and post-MDA surveillance).

### STH

For STH later-stage control programs, no single-test option is currently validated for use as an alternative to the current Kato-Katz method. Molecular tests for parasite nucleic acid markers show the most promise to meet technical performance requirements for next-generation assays. Both lab-based and field-deployable molecular test options need to be considered. Stakeholders have indicated a preference for immunoassay RDTs that assess specific antigens or antibodies for a complete set of STH biomarkers, because this will provide a relatively low-cost, simple, and familiar format, assuming that the technical performance requirements can be met. However, because the availability and validation of these biomarkers is still limited, this represents a long-term and high risk solution.

Investments are needed now to ensure continued progress so that new tools become available. In this early stage, a portfolio-based approach will help to reduce risk while advancing test development across a variety of formats and platforms. Because copromicroscopy methods remain critical for surveillance, investments should also be considered for developing new sample collection, transport, or processing tools or for improving access to current technologies that could enhance performance and usability of current diagnostic tests.

**Top Five Papers**

1. Pullan RL, Smith JL, Jasrasaria R, Brooker SJ. Global numbers of infection and disease burden of soil transmitted helminth infections in 2010. Parasit Vectors 2014;7:37.
2. Bergquist R, Johansen MV, Utzinger J. Diagnostic dilemmas in helminthology: what tools to use and when? Trends Parasitol 2009 Apr;25(4):151–6.
3. Solomon AW, Engels D, Bailey RL, Blake IM, Brooker S, Chen JX, et al. A diagnostics platform for the integrated mapping, monitoring, and surveillance of neglected tropical diseases: rationale and target product profiles. PLoS Negl Trop Dis 2012;6(7):e1746.
4. World Health Organization. Schistosomiasis: progress report 2001–2011, strategic plan 2012–2020.
5. Weerakoon KG, Gobert GN, Cai P, McManus DP. Advances in the Diagnosis of Human Schistosomiasis. Clin Microbiol Rev 28(4):939–967. 2015.

**Key Learning Points**

- As control and elimination programs realign to meet London Declaration goals, the requirements for diagnostics change.
- Better performing, more easily deployed diagnostic tools may support decisions on appropriate allocation of scarce drug resources.
- The current gold-standard microscopy techniques are operationally challenging and do not perform adequately, especially in lower prevalence areas.
- ES parasite antigen tests—if available—can provide the right combination of performance and deployability.
- Molecular testing provides excellent performance, but operational challenges must be overcome.

## Supporting Information

S1 Table Biomarker landscape for schistosomiasis. (DOCX)

S2 Table Diagnostic landscape for schistosomiasis. (DOCX)

S3 Table Methods for detecting STH. (DOCX)

S4 Table Biomarkers and diagnostic technologies for detecting STH. (DOCX)
