# Supplementary material for: Diagnostic Tests to Support Late-Stage Control Programs for Schistosomiasis and Soil-Transmitted Helminthiases
Source: PLoS Negl Trop Dis. 2016 Dec 22;10(12):e0004985. doi: 10.1371/journal.pntd.0004985 (PMC5179049; doi:10.1371/journal.pntd.0004985)
Supplement: S1 Table — (DOCX) [file pntd.0004985.s001.docx]

| **S1 Table. Biomarker Landscape for Schistosomiasis** | | | | | | | | |
| --- | --- | --- | --- | --- | --- | --- | --- | --- |
| **Biomarker** | **Surveillance Measure** | **Description of Priority Candidates** | **Sample Type** | **Format** | **Use Within Schistosomiasis Diagnostics** | **Stage of Product Development** | **Prioritized Use Case** | **Recommendations** |
| **Clinical** | Out of scope (see report text) | | | | | | | |
| **Schistosome Nucleic Acid** | - Infection | DNA   - Dra1 121-bp tandem repeat: genus specific, highly repeated (abundant), both parasite associated (stool, urine) and cell free (plasma, urine) - SSU rRNA gene, ITS-2, 28S rRNA gene, cox1 gene: species specific, cell-free abundance uncharacterized   RNA   - Micro RNA (miRNA223): murine data only, human applications unproven, persistence low, sample type unclear | - Stool, urine, blood | - Lab- or field-based molecular test | - Any research-grade PCR system - Targeted applications have not been qualified. - POC applications have not been qualified | - No commercial diagnostic products available - Limited field demonstrations of research tools - A few RT-PCR reagent kits have been introduced for research only and veterinary use | - MDA stopping - Post-MDA surveillance | **NOT PRIORITIZED AT THIS TIME**  • **Near Term:** No action needed at this time. Nucleic acid amplification tests (NAATs) are judged to be too far upstream in the product development process at this time to prioritize over circulating antigen tests.   • **Long term:** Track progress and landscape of new commercial test options. Development of evaluation of NAATs with lower cost and complexity may be considered if circulating antigen test development stalls. Dra1 121-bp tandem repeats is probably best probe candidate if further development becomes a priority. Multiplexing of NAATs with other neglected tropical diseases (most notably STH) may become a priority as tools for those diseases are developed, and that may be easier with a NAAT than with an antigen detection immunoassay |
| **Schistosome Antigens** | - Infection | - CAA: highly genus specific, suitable for all 3 major human *Schistoma spp*., moderately abundant in blood and urine (independent of ova and parasites), very stable - CCA: fairly genus specific, sensitive only for *S. mansoni*, moderately abundant in blood and urine (independent of ova and parasites) - SEA: complex, unpurified antigen preparation associated with presence of ova in sample - PGM, RAD23: newly discovered (2015) by proteomics, relatively uncharacterized | - Blood, urine, stool, saliva | - Antigen detection immunoassay (RDT, ELISA, etc.) | - Any ELISA platform (research or commercial) - Any RDT (for best limit of detection, a reader may be required): CCA commercialized by Rapid Medical Diagnostics (Pretoria, RSA), CAA-UCP in development by IMS/LUMC, Veritor CAA in development by Becton Dickenson (BD) (San Diego, USA) - SEA used in elimination in Egypt from TBI research product | - Commercial products available (CCA-RDT) - New products in development (CAA RDTS) - Research “home-brews” common - Recent discovery of new Ag - Field demon-strations of newly developed CAA UCP RDT | - MDA stopping - Post-MDA surveillance | **PRIORITIZED**  • **Near term:** Continue to work with BD and IMS/LUMC to develop a high-sensitivity CAA RDT that meets minimal TPP attributes. Continue to support SCORE and others in field evaluation of CCA in *S. mansoni* endemic regions.  • **Long term:** Support further improvements of LOD down to the ideal TPP target through development of pre-concentration technologies (on or off strip) for CAA RDTs. Support multiplexing of CAA with schistosome antibodies (for the post-elimination use case) or with other neglected tropical diseases (especially STH) |
| **Host Antibody** | - Exposure | - α-SmCTF: detected with complex and relatively uncharacterized Ag reagent, SmCTF Ag is cheaper and easier to obtain than ova- and parasite-derived material, not capable of differentiating active infection from past exposure - α-AWA, α-SEA, α-CEF6, α-SBgA (and others): detected with complex and relatively uncharacterized Ag reagent, Ag are obtained from homogenized parasites and snails with little or no purification, not capable of differentiating active infection from past exposure | - Blood | - Antibody detection immunoassay (RDT, ELISA, etc.) - Note: IHA, COPT, DDIA, and other older techniques are out of scope (see text) | - Any ELISA platform (research or commercial) - Any RDT; SmCTF is under active development at Omega Diagnostics with BioGlab (both UK). Previous development at Vision Diagnostics with BioGlab | - New products in development (α-SmCTF RDT) - Research “home brews” are common - Limited field demonstrations of α-SmCTF RDT | - Post-MDA surveillance | **NOT PRIORITIZED AT THIS TIME**  • **Near term:** Track progress and maintain communication with Omega and BioGlab. Support (without investment) development of α-SmCTF RDT and field studies that establish utility.   • **Long term:** Track progress and landscape of new commercial test options. If α-SmCTF proves to be a useful analyte, a biplex with CAA on an RDT could be useful to create the most-accurate post-elimination tool. |

Abbreviations: IHA = indirect hemagglutination assay; COPT = circumoval precipitin test; DDIA = dipstick dye immunoassay; SmCTF = *S. mansoni* cercarial transformation fluid
