# Supplementary material for: Diagnostic Tests to Support Late-Stage Control Programs for Schistosomiasis and Soil-Transmitted Helminthiases
Source: PLoS Negl Trop Dis. 2016 Dec 22;10(12):e0004985. doi: 10.1371/journal.pntd.0004985 (PMC5179049; doi:10.1371/journal.pntd.0004985)
Supplement: S2 Table — (DOCX) [file pntd.0004985.s002.docx]

| **S2 Table: Diagnostic Landscape for Schistosomiasis** | | | | | | | | |
| --- | --- | --- | --- | --- | --- | --- | --- | --- |
| **Company** | **Diagnostic** | **Biomarker** | **Surveillance Measure** | **Availability** | **Description** | **Current Status for Use in Schistosome Surveillance** | **Advantages** | **Limitations** |
| **Multiple Suppliers** | Clinical and older microscopic and serological tests | Out of Scope (obsolete or inaccurate, see text) | | | | | | |
| **Multiple Research Laboratories** | Many types | Many academic research projects have created “home brew” tests for all biomarker and sample types. These are not called out in this technology landscape unless they display a high potential for advancement into a commercial product in the next five years. See text and bibliography for further reference. | | | | | | |
| **Becton Dickenson** | Veritor ICS RDT | CAA | Infection | In development  (awaiting funding) | - Multiplexed ICS and POC reader - Enhanced gold detector conjugate - On strip positive and negative controls used for quantitative signal correction algorithms - Uses LUMC’s very high affinity antibody reagents | - In development - Feasibility studies complete | - Proven platform - Experienced large diagnostic company who already has CLIA waived products on the platform - Feasibility studies suggest LOD better than any product currently sold or used - Inventors of CAA test are involved | - Closed platform - May not get LOD to ideal TPP target without pre-concentration - User acceptance in target market not yet demonstrated |
| **Intelligent Material Solutions** | ICS RDT | CAA | Infection | In development | - UCP detector conjugates—made by Intelligent Material Solutions - Cognate reader - Uses LUMC’s very high affinity antibody reagents | In development | - UCP detection system is part of LUMC demonstration of the only tests that has ever reached TPP ideal targets - Inventors of CAA test are involved | - UCP reader is only useful for tests that use/need the UCP conjugate - Plans for reaching ideal TPP LOD targets are unclear - User acceptance in target market not yet demonstrated |
| **Rapid Medical Diagnostics** | ICS RDT | CCA | Infection | Commercialized | - Visually read ICS - Gold detector conjugate | - Extensive field validation studies - Findings recently summarized in Cochrane Review (see text) | - Easy and familiar to perform - Uses urine sample instead of stool - Good performance for *S. mansoni* - May supplant Kato-Katz for mapping | - Not sensitive for other *Schistoma spp.* - Low worm burdens give trace results that are hard to score - Perceived as too expensive by some key opinion leaders |
| **Omega** | ICS RDT | α-SmCTF | Exposure | In development | - Based on BioGlab’s SmCTF antigen reagent - Visually read - SmCTF appears useful for some use cases, but validation is ongoing | - In development - Limited field studies | - Antigen reagent is relatively cheap/ fast/easy to produce - Limited field studies are positive to date | - Ability to scale up is still undemonstrated - Antibody tests are generally only useful for a post-elimination use case |
| **Ng-Biotech** | ICS RDT | Antibody to Proprietary  Ag | Exposure | In development | - Unique Multiplex capability - Can be quantitative (using multiplex) - Read visually or with cloud enabled reader. | In development | Proprietary—performance and ability to meet product claims still unknown | - Proprietary—performance and ability to meet product claims still unknown - Antibody tests are generally only useful for a post-elimination use case |
| **Shanghai ZJ Bio-Tech Co** | Liferiver RT-PCR reagent kit for use with many common RT-PCR machines | Target not specified in package insert | Infection | Commercialized | - Chinese-made research only RT-PCR kit for *S. japonicum* only - Typical RT-PCR kit components - FAM™ channel detection - Claimed LOD: 5×103 copies/ml | Research only | Little information available | - Research only, for *S. japonicum* only - Requires extraction - Diagnostic value not established - Not POC |
| **Vacunek** | RT-PCR reagent kit | ? | Infection | Commercialized | - Veterinary product - Little information available | Veterinary product; not qualified for human use | Little information available | - Veterinary product—not clear if it is sensitive to human-infecting *Schistoma spp.* - Requires extraction - Diagnostic value not established - Not POC |
| **Abcam, Genway, Bordier Affinity Products, etc.** | ELISA kit | Antibody to Proprietary  Ag | Exposure | Commercialized | - Typical research-only indirect ELISA kit - HRP-TMB detection - Ag from *S. mansoni* | Research only | Little information available | Little information available |
| **Diagnositc Automation/ Cortez Diagnositics, etc.** | ELISA kit | Antibody to Proprietary  Ag | Exposure | Commercialized | - Typical research-only sandwich ELISA kit - HRP-TMB detection - “Recombinant schistosome Ag” - Qualitative test with stop solution | Research only | Little information available | Little information available |
| **IVD Research, etc.** | ELISA kit | Antibody to Proprietary  Ag | Exposure | Commercialized | - Little information available | Research only | Little information available | Little information available |
