# Supplementary material for: Diagnostic Tests to Support Late-Stage Control Programs for Schistosomiasis and Soil-Transmitted Helminthiases
Source: PLoS Negl Trop Dis. 2016 Dec 22;10(12):e0004985. doi: 10.1371/journal.pntd.0004985 (PMC5179049; doi:10.1371/journal.pntd.0004985)
Supplement: S3 Table — (DOCX) [file pntd.0004985.s003.docx]

**S3 Table.** Methods for detecting soil-transmitted helminthes.

| **Company** | **Diagnostic** | **Biomarker** | **Surveillance Measure** | **Availability** | **Description** | **Current Status for use in STH Surveillance** | **Advantages** | **Limitations** |
| --- | --- | --- | --- | --- | --- | --- | --- | --- |
| WHO | Kato-Katz | Eggs | Infection | Developed | - Microscopic counting of eggs from 41.7 mg stool - Examination involves the use of fresh stool specimens on a glass slide and covered with a cellophane pre-soaked in glycerin | - Approved for use by WHO - Current methodology used for informing control program decisions | - Gold standard - Quantitative - Field-deployable - Relatively inexpensive - Good sensitivity for moderate- or heavy-intensity infection - Detects active infection and intensity of infection | - Performance limitations at low prevalence (Low PPV) - Subjective interpretation - Reproducibility - Specialized training - Dependent on egg-shedding - Time sensitive |
| University of Sydney | McMaster | Eggs | Infection | Developed | - Microscopic counting of eggs from 2-g stool - Examination involves the use of fresh stool specimens on a counting chamber that allows examination of known volume of fecal suspension - Method include flotation of eggs | - Approved for use by WHO - Standard reference method for evaluating drug efficacy in veterinary parasitology and was evaluated for human helminths - Alternative methodology used for monitoring MDA | - Quantitative - Field-deployable - Relatively inexpensive - Good sensitivity for moderate- or heavy-intensity infection - Detects active infection and intensity of infection - Easier to read than Kato-Katz | - Performance limitations at low prevalence (Low PPV) - Subjective interpretation - Specialized training - Dependent on egg-shedding - Time sensitive - Requires flotation solution - Buoyancy of eggs may vary |
| University of Naples, Italy | Mini-FLOTAC | Eggs | Infection | Developed | - Microscopic counting of eggs from 2-g stool - Uses a sampling device also used for flotation of eggs - Uses either fresh or preserved stool specimens | - Approved for use by WHO - Alternative methodology used for informing control program decisions | - Quantitative - Field-deployable - Relatively inexpensive - Good sensitivity for moderate- or heavy-intensity infection - Detects active infection and intensity of infection - Not time sensitive | - Performance limitations at low prevalence (Low PPV) - Subjective interpretation - Specialized training - Dependent on egg-shedding - Requires flotation and formalin solutions - Equipment and reagents not readily available in endemic countries |
| University of Naples, Italy | FLOTAC | Eggs | Infection | Developed | - Microscopic counting of helminth eggs - Uses a FLOTAC device with flotation chambers and allows up to 1-g stool to be prepared for analysis - Uses either fresh or preserved stool specimens | - Research use - Developed for use in diagnosing parasites in animals and humans | - Quantitative - Relatively inexpensive - Good sensitivity for moderate- or heavy-intensity infection - Detects active and intensity of infection - Not time sensitive | - Performance limitations at low prevalence (Low PPV) - Subjective interpretation - Specialized training - Dependent on egg-shedding - Requires centrifuge, as well as flotation and formalin solutions - Equipment and reagents not readily available in endemic countries |
|  | FECT (formalin-ether concentration technique) | Eggs | Infection | Developed | - Microscopic counting of helminth eggs - Concentrate the parasite in the sediment from 1-g stool using solutions with lower specific gravity than the parasite - Uses either fresh or preserved stool specimens | - Methodology used by CDC to identify parasitic diseases of public health concern | - Good sensitivity for moderate- or heavy-intensity infection - Detects active and intensity of infection - Not time sensitive | - Performance limitations at low prevalence (Low PPV) - Subjective interpretation - Specialized training - Dependent on egg-shedding - Requires centrifuge, as well as sedimentation and formalin solutions - Equipment and reagents not readily available in endemic countries |
| NIH | Multi-parallel real-time PCR | DNA | Infection | Research and development | - Amplification technology: TaqMan probe-based real-time PCR - Target for STH: ribosomal DNA - Lab-based NAAT developed using ABI 7900HT Fast Real-Time PCR System - Adapted for use with stool samples | - Homebrew assay currently for research use - Validated by TFGH - Potential for use by control programs contingent upon results of evaluation | - Good accuracy even at low prevalence - Detects active infection - High-throughput platform available - Ease in test interpretation | - Not WHO-approved - Cost - Infrastructure requirements - High-complexity - Needs separate sample extraction step - Needs thermocycler |
| Leiden University Medical Center, The Netherlands | Multiplex real-time PCR | DNA | Infection | Research and development | - Amplification technology: TaqMan probe-based real-time PCR - Target for STH: ribosomal DNA - Lab-based NAAT developed using ABI 7900HT Fast Real-Time PCR System - Adapted for use with stool samples | - Homebrew assay currently for research use - Validated by TFGH - Potential for use by control programs contingent upon results of evaluation | - Good accuracy even at low prevalence - Detects active infection - High-throughput platform available - Ease in test interpretation | - Not WHO-approved - Cost - Infrastructure requirements - High-complexity - Needs separate sample extraction step - Needs thermocycler |
| Smith College | Multi-parallel real-time PCR | DNA | Infection | Research and development | - Amplification technology: TaqMan probe-based real-time PCR - Target for STH unknown but identified by NGS - Lab-based NAAT system - Adapted for use with stool samples | - Homebrew assay currently for research use for STH - Validated by TFGH - Potential for use by control programs contingent upon results of evaluation | - Good accuracy even at low prevalence - Detects active infection - High-throughput platform available - Ease in test interpretation - Most sensitive PCR assay than the two PCR assays validated | - Not yet WHO-approved - Cost - Infrastructure requirements - High-complexity - Needs separate sample extraction step - Needs thermocycler |
