# Supplementary material for: Diagnostic Tests to Support Late-Stage Control Programs for Schistosomiasis and Soil-Transmitted Helminthiases
Source: PLoS Negl Trop Dis. 2016 Dec 22;10(12):e0004985. doi: 10.1371/journal.pntd.0004985 (PMC5179049; doi:10.1371/journal.pntd.0004985)
Supplement: S4 Table — (DOCX) [file pntd.0004985.s004.docx]

**S4 Table**. Biomarkers and diagnostic technologies for detecting soil-transmitted helminthes.

| **Biomarkers** | **Technologies** | **Specific target** | **Pros** | **Limitations** | **Conclusions** |
| --- | --- | --- | --- | --- | --- |
| Parasite eggs in stool | Microscopy   - Kato-Katz - Mini-FLOTAC - McMaster | - Eggs | - Gold standard - Quantitative - Cheap, simple to perform - No sophisticated equipment needed other than microscope - Detects active infection and intensity of infection | - Not very sensitive - Dependent on egg-shedding, some eggs are missed - Specificity depends on microscopist - Cannot distinguish hookworm eggs from each other | - Widely used - Useful in high prevalence settings (mapping and impact monitoring) - Not sensitive enough for MDA-reduction and post-MDA control programs |
| Parasite proteins in stools | Immunoassay-based test | - Excretory/ secretory proteins - Somatic antigens - Adult worm or larval proteins | - Detects active infections - Independent of egg-shedding - Field deployable - Easy to use, minimal training required - Low cost | - Limited studies as diagnostic tool - Immunoreagents for main species are in different development stages - Worm antigens may not be present in sufficient amount in stool to allow detection - Biomarkers for all 4 species at different stages of validation/discovery | - Test not currently available - Depending on reagent availability and performance, maybe useful for mapping, impact monitoring and MDA reduction decision - *Preliminary works showed promising result* |
| Parasite nucleic acid in stool | Molecular tests | - Ribosomal DNA - Mitochondrial DNA | - Detects current infection - Highly sensitive and specific - Qualitative and quantitative - Multiplex-capable | - Equipment and reagent cost, and availability - Requires trained technician - *May be dependent on egg-shedding* - May need separate sample extraction step | - PCR methods exist and validated, but results are not yet out - Isothermal amplification method not currently available, but may overcome PCR limitations |
| Antibodies against worm antigens in serum | Immunoassay-based test | - IgG | - Independent of egg-shedding - Uses blood instead of stool - Some antibody types (IgG4) correlated with intensity of infection - Field deployable - Easy to use, minimal training required - Low cost | - Limited studies as diagnostic tool - Does not distinguish current from past exposure as host antibodies persist after treatment | - Test not currently available - Depending on reagent availability and performance, maybe useful in post-MDA surveillance - *Can be easily integrated with other disease dx* |
